# Parishin A Inhibits Oral Squamous Cell Carcinoma via the AKT/mTOR Signaling Pathway

**DOI:** 10.3390/ph17101277

**Published:** 2024-09-26

**Authors:** Lei Ma, Zhibin Liu, Eungyung Kim, Ke Huang, Chae Yeon Kim, Hyeonjin Kim, Kanghyun Park, Woo-Sung Kwon, Sang In Lee, Yong-Gun Kim, Youngkyun Lee, So-Young Choi, Haibo Zhang, Myoung Ok Kim

**Affiliations:** 1 Department of Animal Science and Biotechnology, Research Institute for Innovative Animal Science, Kyungpook National University, Daegu 37224, Republic of Korea; 2 Department of Oral and Maxillofacial Surgery, School of Dentistry, University of Texas Health Science Center at San Antonio, San Antonio, TX 78229, USA; 3 Department of Periodontology, School of Dentistry, Kyungpook National University, Daegu 41566, Republic of Korea; 4 Department of Biochemistry, School of Dentistry, Kyungpook National University, Daegu 41940, Republic of Korea; 5 Department of Oral & Maxillofacial Surgery, School of Dentistry, Kyungpook National University, Daegu 41566, Republic of Korea; 6 College of Pharmacy, Henan University of Chinese Medicine, Zhengzhou 450046, China

**Keywords:** EMT, oral squamous cell carcinoma, parishin A, PI3K/AKT/mTOR pathway, proliferation

## Abstract

Background: Oral squamous cell carcinoma (OSCC) is an aggressive cancer with limited treatment options. Parishin A, a natural compound derived from *Gastrodia elata*, possesses multiple therapeutic properties. However, its effects on OSCC remain unexplored. Purpose: This study explores the anti-cancer potential of Parishin A on OSCC and its mechanisms. Methods: OSCC cell lines YD-10B and Ca9-22 were treated with varying Parishin A concentrations. Cell viability was detected using the CCK-8 assay, and colony formation was evaluated in agarose gel. Migration and invasion ability were assessed through wound healing and Matrigel invasion assays. The protein expression levels involved in the PI3K/AKT/mTOR signaling pathway and epithelial–mesenchymal transition (EMT) markers were examined via Western blotting. Results: Parishin A inhibited OSCC cell viability in both dose- and time-dependent manners, with significant reductions at 20, 40, 60, and 80 μM, without affecting normal human gingival fibroblasts. Colony formation decreased substantially at ≥40 μM higher Parishin A concentrations in a dose-dependent manner. Also, migration and invasion assays showed significant suppression by Parishin A treatment concentration ≥40 μM in a dose-dependent manner, as evidenced by decreased wound closure and invasion. Western blot analyses revealed increased E-cadherin levels and decreased N-cadherin and vimentin levels, suggesting EMT inhibition. Parishin A also decreased the phosphorylation levels of PI3K, AKT, and mTOR. Conclusion: Collectively, these findings support the potential of Parishin A as an anti-OSCC agent.

## 1. Introduction

Oral cancer encompasses malignancies arising from various tissues within the oral cavity, including the lips, tongue, gingiva, floor of the mouth, buccal mucosa, and hard palate. Each of these tissues can give rise to different types of oral squamous cell carcinoma (OSCC), which together represent the most common form of oral cancer [1]. OSCC is a common cancer in the oral cavity, accounting for over 90% of all oral malignancies [2]. This disease is marked by its tendency to aggressively invade nearby tissues and its high likelihood of spreading to other parts of the body, leading to considerable suffering and death [3]. Oral cancer is one of the ten most common cancers globally, though its incidence varies significantly across different regions. This variation is largely influenced by factors such as alcohol consumption, tobacco use, and human papillomavirus (HPV) infection [4]. Despite significant advances in treatment, including surgery, radiation therapy, and chemotherapy, the prognosis for OSCC patients remains poor, particularly in advanced stages [5]. Therefore, there is an urgent need for novel therapeutic agents that can effectively target OSCC cells with minimal side effects.

**Figure 1:**
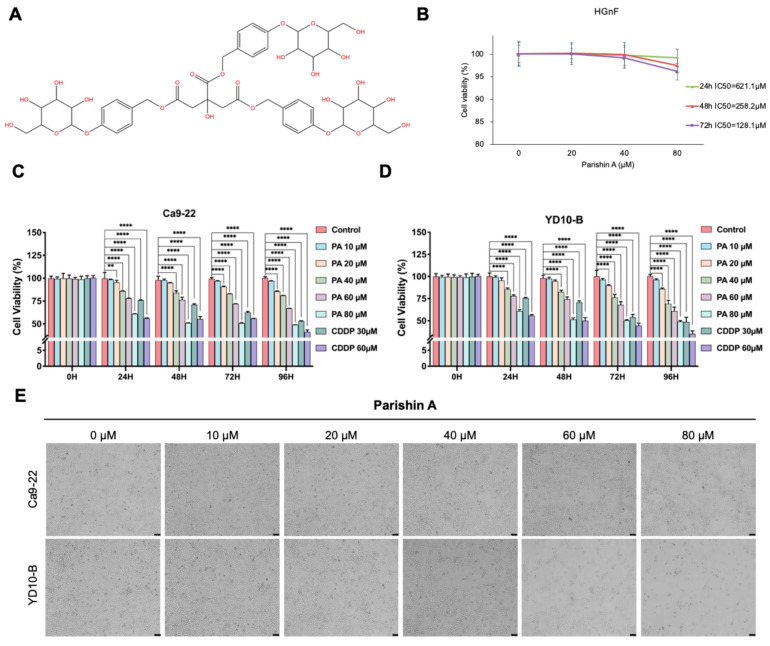
Parishin A inhibits OSCC cell growth. (**A**) Chemical structure of Parishin A. (**B**) Cell viability of normal human gingival fibroblasts (HGnFs) after treatment with 0 μM, 20 μM, 40 μM, and 80 μM Parishin A for 24, 48, and 72 h. (**C**,**D**) Cell viability of OSCC cell lines YD-10B and Ca9-22 after treatment with 0 μM, 10 μM, 20 μM, 40 μM, 60 μM, and 80 μM Parishin A for 0, 24, 48, 72, and 96 h, determined by the CCK-8 assay. (**E**) Morphological changes in YD-10B and Ca9-22 cells after treatment with 0 μM, 10 μM, 20 μM, 40 μM, 60 μM, and 80 μM Parishin A for 0, 24, 48, 72, and 96 h, observed under a light microscope (magnification, 100×). Data are shown as means ± SD from three independent experiments, each with triplicate samples. Asterisks indicate significant inhibition (** *p* < 0.01, **** *p* < 0.0001).

Natural compounds have garnered significant attention in cancer research due to their potential efficacy and lower toxicity compared to conventional chemotherapeutics [6]. Gastrodia elata is a well-known medicinal herb used in traditional medicine across various countries in East Asia, including China, Korea, and Japan. Its therapeutic benefits are well documented, encompassing neuroprotective, anti-inflammatory, and antioxidant effects [7]. Parishin A (the chemical structure shown in Figure 1A), a bioactive compound extracted from *Gastrodia elata*, has traditionally been used for its anti-inflammatory and neuroprotective properties. Recent studies have shown that Parishin A possesses significant anti-inflammatory effects, aiding conditions such as tendinopathy [8]. It also provides neuroprotective benefits, particularly for brain disorders and long-term potentiation deficits [9]. Furthermore, Parishin A can enhance Klotho expression, helping to mitigate vascular endothelial cell aging and delay vascular senescence [10]. Parishin markedly lowers the levels of aging-related biomarkers such as GDF15, IL-6, and p16 Ink 4 a. It also enhances the balance of the intestinal microbiota and metabolome, while alleviating pathological changes in cardiopulmonary tissues [11]. Parishin A also exhibits antiviral activity by inhibiting Zika virus entry and offers cardioprotective effects against cardiac aging [12]. Based on recent studies, Parishin A and its related compounds have shown promising effects on various aspects of cancer treatment. Specifically, Parishin has been found to moderately reduce the activity of the multidrug resistance (MDR) efflux pump in lymphoma cells, which is crucial for overcoming chemotherapy resistance. Additionally, Parishin-related compounds have demonstrated the ability to significantly enhance antibody-dependent cellular cytotoxicity (ADCC), which is vital for improving immune responses against cancer cells. These findings suggest that Parishin compounds, including Parishin A, may contribute to cancer treatment not only through direct cytotoxic effects but also by modulating the immune system to enhance anti-cancer activities [13]. Furthermore, the anti-cancer potential of Parishin A may extend to its effects on other cancers, such as hepatocellular carcinoma (HCC), where G. elata derivatives have been shown to exhibit immunomodulatory properties that enhance the effectiveness of immune cells in targeting cancer cells [14]. However, its specific effects on OSCC have not been extensively studied.

This study aimed to explore the anti-cancer properties of Parishin A in OSCC. We utilized the OSCC cell lines YD-10B and Ca9-22 as in vitro models to evaluate how Parishin A affects cell viability and metastatic behavior. Understanding the mechanisms underlying the effects that Parishin A exerts could provide valuable insights, enabling the development of new therapeutic strategies for OSCC.

## 2. Results

### 2.1. Parishin A Inhibits OSCC Cell Growth

Figure 1A shows the chemical structure of Parishin A. To assess the impact on cell viability, the OSCC cell lines YD-10B and Ca9-22 were exposed to escalating concentrations of Parishin A (ranging from 0 to 80 µM) over various time periods (24, 48, 72, and 96 h). Figure 1B presents the cell viability of HGnF treated with 0, 20, 40, and 80 μM Parishin A for 24, 48, and 72 h. These results indicate that Parishin A does not significantly affect the viability of HGnF cells, suggesting no potential toxicity to normal oral cells. Figure 1C,D show the cell viability of Ca9-22 and YD-10B OSCC cells, respectively, after treatment with 0, 10, 20, 40, 60, and 80 μM Parishin A for 0, 24, 48, 72, and 96 h, as determined by the CCK-8 assay. Both figures demonstrate a dose- and time-dependent decrease in cell viability. Significant reductions in viability were observed at concentrations of 40 μM and higher, particularly after 48, 72 and 96 h of treatment, which were similar to the positive control groups. Parishin A demonstrates significant effectiveness in suppressing the growth of OSCC cells. Figure 1E illustrates the morphology of YD-10B and Ca9-22 cells treated with 0, 10, 20, 40, 60, and 80 μM Parishin A for 0, 24, 48, 72, and 96 h, as observed under a light microscope (magnification, 100×). The images show noticeable morphological changes, including cell shrinkage and detachment, particularly at higher concentrations and longer exposure times, indicating cell death and reduced proliferation. Together, these results indicate that Parishin A effectively reduces the growth and survival of OSCC cells in a manner that is both dose-dependent and time-dependent, without showing any toxicity to normal oral cells.

### 2.2. Colony Formation Inhibition by Parishin A

**Figure 2:**
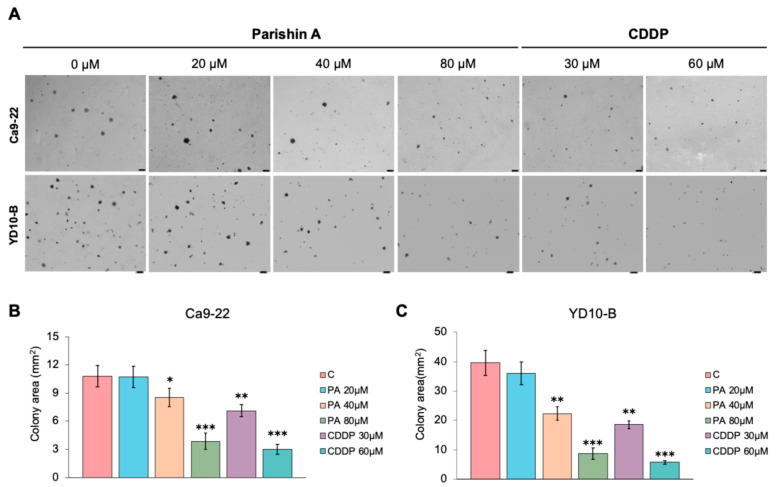
Parishin A inhibits OSCC colony formation abilities. (**A**) Effect of Parishin A on anchorage-independent growth of Ca9-22 cells and YD-10B cells. (**B**) Representative images of colonies formed by Ca9-22 cells, with quantification performed using the Image-Pro PLUS (v.6) software. (**C**) Representative images of colonies formed by YD-10B cells, with quantification performed using the Image-Pro PLUS (v.6) software. Data are shown as means ± SD from three independent experiments, each with triplicate samples. Asterisks indicate significant inhibition (* *p* < 0.05, ** *p* < 0.01, and *** *p* < 0.001) of colony formation by Parishin A.

Figure 2 presents the effect of Parishin A on the colony-forming ability of Ca9-22 and YD-10B cells, treated with increasing concentrations of Parishin A (20, 40, and 80 µM), as well as the CDDP at 30 and 60 µM. The cells were cultured in agarose for 14 days to evaluate colony formation. Figure 2A shows representative colony formations in Ca9-22 and YD-10B cells after treatment. As the concentration of Parishin A increases, there is a noticeable reduction in both the number and size of colonies for both cell lines. This decrease is more pronounced at higher concentrations of Parishin A (80 µM), which shows an inhibition comparable to that of CDDP (60 µM). Quantitative analysis of the colony area for Ca9-22 cells (Figure 2B) reveals a significant decrease in colony size with Parishin A treatments at 40 µM and 80 µM, with the latter concentration showing the strongest inhibitory effect. This reduction is similar to the colony inhibition observed with CDDP (60 µM). For YD-10B cells (Figure 2C), a significant reduction in colony area is observed with Parishin A treatments at concentrations of 40 µM and higher, with a strong inhibitory effect at 80 µM, closely resembling the results obtained with CDDP. These results confirm that Parishin A effectively inhibits the colony formation of OSCC cells in a dose-dependent manner, with its effects being comparable to those of the reference drug CDDP at higher concentrations.

### 2.3. Parishin A Suppresses Migration and Invasion in OSCC Cells

**Figure 3:**
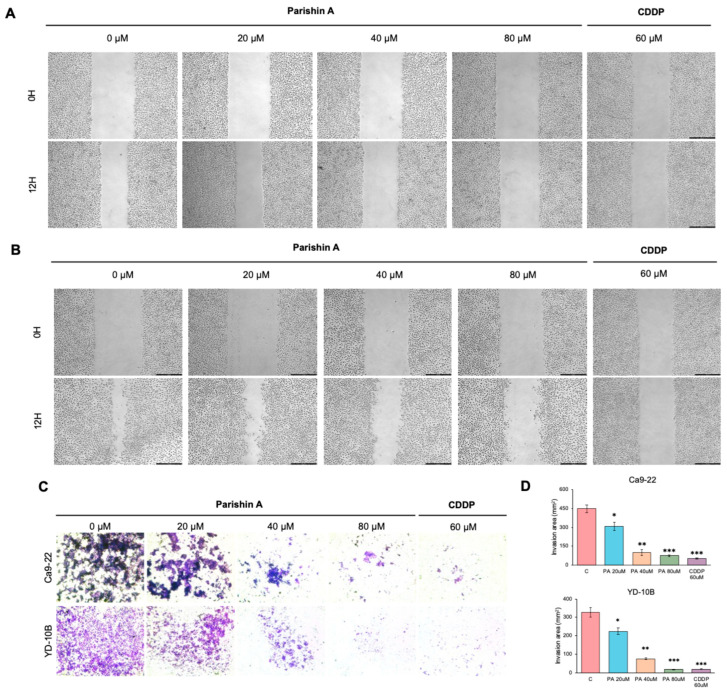
Parishin A suppresses the migration and invasion of OSCC cells. (**A**) Migration of Ca9-22 cells treated with Parishin A, CDDP or DMSO control at 0 and 12 h post-wounding. (**B**) Migration of YD-10B cells treated with Parishin A, CDDP or DMSO control at 0 and 12 h post-wounding. (**C**) Representative images of Ca9-22 and YD-10B cells that invaded through Matrigel after treatment with Parishin A, CDDP or DMSO control. (**D**) Quantification of the invaded cell area for each treatment condition. * *p* < 0.05, ** *p* < 0.01, and *** *p* < 0.001 compared with 0 μM.

**Figure 4:**
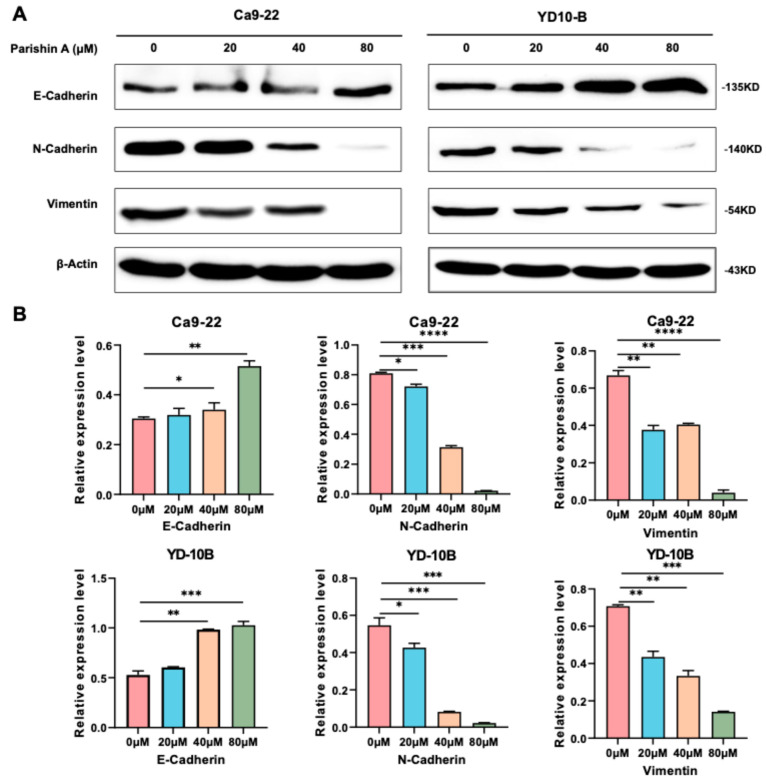
Parishin A suppresses the EMT process in OSCC cells. (**A**) Western blot analysis of protein expression levels related to EMT in Ca922 and YD10B cells treated with Parishin A. Cells were treated with varying concentrations of Parishin A (0, 10, 20, 40, and 80 μM) for 48 h. (**B**) Quantification of the protein expression levels of Western blot for different markers. Data are shown as means ± standard deviation of values from three independent experiments each with triplicate samples. * *p* < 0.05, ** *p* < 0.01, *** *p* < 0.001, and **** *p* < 0.0001 compared with 0 μM.

In this study, we examined how Parishin A influences the migration and invasion of OSCC cells through wound healing and Matrigel invasion assays, and we evaluated its effects on the expression levels of relevant proteins. In Figure 3A, images of Ca9-22 cells treated with Parishin A at concentrations of 0, 20, 40, and 80 μM, taken 0 and 12 h after wounding, show a significant reduction in wound closure compared to the DMSO control, indicating inhibited cell migration. Additionally, the CDDP group, used as a positive control, exhibited a similar reduction in wound closure, confirming the effectiveness of the assay. Similarly, Figure 3B presents images of YD-10B cells treated with the same concentrations of Parishin A, which also exhibit markedly reduced migration, with less wound closure observed compared to controls. The CDDP-treated YD-10B cells also show a comparable reduction in migration, demonstrating that Parishin A’s effect is comparable to that of the positive control. Figure 3C provides representative images of Ca9-22 and YD-10B cells treated with Parishin A at the same concentrations, assessed by the Matrigel invasion assay. The number of invading cells significantly decreases with increasing concentrations of Parishin A, highlighting its inhibitory effect on cell invasion. Similarly, the CDDP-treated group also shows a marked reduction in cell invasion, consistent with its role as an established therapeutic agent. Quantitative analysis of the invasion area, shown in Figure 3D, indicates a significant reduction for both cell lines treated with Parishin A compared with the controls. Additionally, Figure 4A,B illustrate the Western blot analysis of protein expression levels related to cell migration and invasion, including E-cadherin, N-cadherin, vimentin, and β-actin as a loading control. The results demonstrate that Parishin A treatment increases E-cadherin expression and decreases N-cadherin and vimentin expression in both cell lines, suggesting a suppression of epithelial–mesenchymal transition (EMT). These findings indicate that Parishin A effectively suppresses both the migration and invasion of OSCC cells by inhibiting the EMT process, highlighting its potential as a therapeutic agent in preventing OSCC metastasis.

### 2.4. Parishin A Inhibits OSCC via the PI3K/AKT/mTOR Signaling Pathway

**Figure 5:**
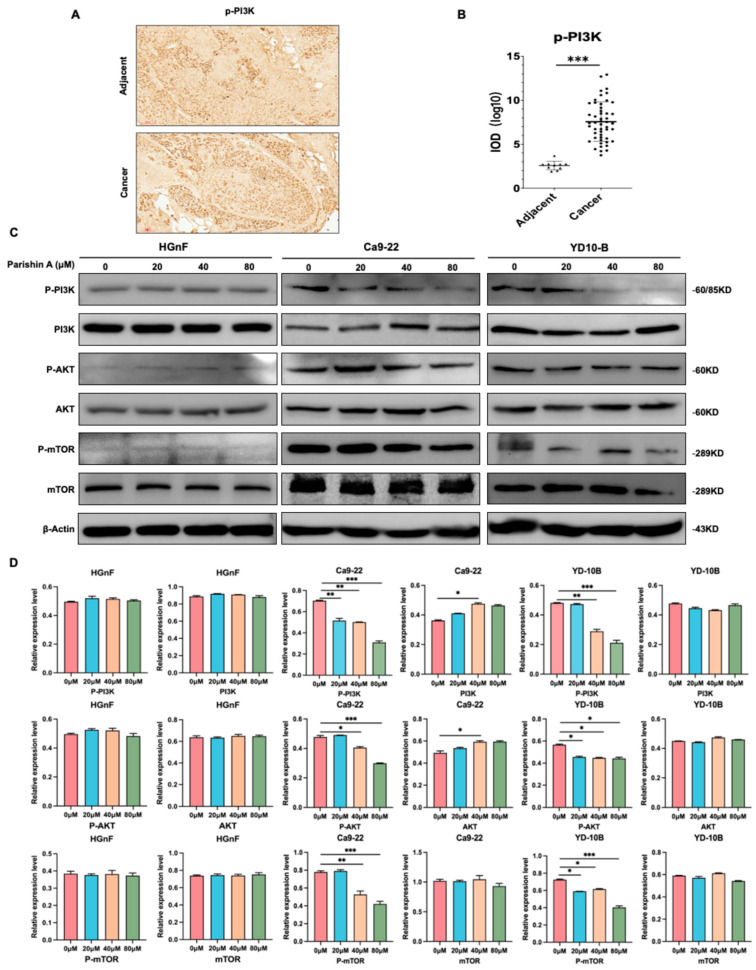
Parishin A inhibits OSCC via the PI3K/AKT/mTOR signaling pathway. (**A**) Expression of p-PI3K in OSCC tissues versus adjacent normal tissues, examined by IHC (100× magnification). (**B**) Quantification of pPI3K expression in OSCC tissues versus adjacent normal tissues. (**C**) Western blot analysis showing the levels of PI3K, pPI3K, AKT, pAKT, mTOR, and pmTOR in HGnF and OSCC cells treated with Parishin A. (**D**) Quantification of the protein expression levels of Western blot for different markers. Data are shown as means ± standard deviation of values from three independent experiments each with triplicate samples. * *p* < 0.05, ** *p* < 0.01, and *** *p* < 0.001 compared with 0 μM.

As illustrated in Figure 5, Parishin A inhibits OSCC by targeting the PI3K/AKT/mTOR signaling pathway, as evidenced by both IHC and Western blot analyses. In Figure 5A, IHC analysis (100× magnification) shows a significant increase in p-PI3K expression in OSCC tissues compared to adjacent normal tissues. This difference in expression is quantitatively confirmed in Figure 5B, where the intensity of p-PI3K is significantly higher in cancerous tissues. Western blot analysis (Figure 5C,D) offered additional understanding of the molecular mechanism driving this effect. The first column represents the protein expression levels in a normal cell line, serving as a baseline for comparison with the other cell lines or treatment conditions. The total PI3K, AKT, and mTOR levels do not differ significantly between the normal and OSCC cell line, showing that PI3K, AKT, and mTOR are present in similar amounts across different cell types. However, compared with treated cancer cells, p-PI3K levels were significantly lower in normal cell lines, while p-AKT and p-mTOR were essentially absent, suggesting that the PI3K pathway is less active in normal cells than in cancer cells, revealing that Parishin A treatment leads to a decrease in the phosphorylation levels of PI3K, AKT, and mTOR, whereas the total levels of these proteins remain unchanged. These results suggest that Parishin A effectively inhibits the PI3K/AKT/mTOR signaling pathway, which is crucial for OSCC cell proliferation and survival. Thus, Parishin A shows potential as a therapeutic agent for targeting and inhibiting key pathways involved in OSCC progression.

## 3. Discussion

OSCC poses a significant health burden due to its high prevalence and aggressive nature, resulting in severe morbidity and mortality [15]. Current treatments, including surgery, radiation, and chemotherapy, often fail to improve the prognosis for patients with advanced OSCC, highlighting the urgent need for new and effective therapies [16]. Natural compounds such as Parishin A, derived from *Gastrodia elata*, offer a promising alternative due to their potential efficacy and reduced toxicity [8]. Parishin A has demonstrated various beneficial properties, such as neuroprotection and anti-inflammatory effects, and emerging evidence suggests it may also possess anti-cancer capabilities [9,14]. Therefore, this study sought to evaluate the effects of Parishin A on OSCC and investigate its potential therapeutic mechanisms.

Cell proliferation and colony formation are fundamental processes in cancer progression [17]. Uncontrolled cell proliferation allows cancer cells to multiply rapidly, leading to the formation of tumors that can invade surrounding tissues [18]. In our study, Parishin A inhibited OSCC cell viability in a dose- and time-dependent manner, with significant reductions observed at concentrations of 40 μM and higher (Figure 1C,D). Previous studies have shown that the natural compound Dioscin can effectively inhibit the proliferation and cloning ability of OSCC, which is similar to our results [19]. However, our study further tested the toxic effects on normal cells. This selective inhibition, without affecting normal human gingival fibroblasts (Figure 1B), underscores the potential of Parishin A as a targeted anti-cancer agent, minimizing the side effects typically associated with conventional chemotherapy. Colony formation in soft agar assays is a hallmark of anchorage-independent growth, a characteristic of malignant cells that contributes to their metastatic potential. Parishin A significantly reduced the colony-forming ability of OSCC cells, highlighting its ability to impair the capacity of cells to grow and survive independently (Figure 2A–C). The comparison between Parishin A and the reference drug, cisplatin, demonstrates that Parishin A, at its highest concentration (80 µM), yields similar inhibitory effects on colony formation to cisplatin at 50 µM. This suggests that while cisplatin is a well-established chemotherapeutic agent, Parishin A can achieve comparable potency, especially at higher doses. The ability of Parishin A to inhibit colony growth at levels similar to cisplatin positions it as a potential alternative therapeutic option, particularly given its lower toxicity profile toward normal cells. Due to its inhibitory effects on both cell proliferation and colony formation, Parishin A could potentially disrupt the early stages of metastasis, which are critical for cancer progression and spread. These promising results provide a solid foundation for designing further experiments with Parishin A, particularly in exploring its potential as a lead compound in anti-metastatic drug development.

EMT is a crucial process in cancer metastasis, during which epithelial cells lose their cell–cell adhesion properties and acquire mesenchymal traits, enhancing the migratory and invasive capabilities [20]. This process is crucial for the spread of cancer cells from the original tumor to distant organs [21]. In our study, Parishin A significantly suppressed the migration and invasion of OSCC cells, as evidenced by decreased wound closure and reduced invasion through Matrigel (Figure 3A–C). Notably, when compared to the reference drug (likely DMSO-treated control or another therapeutic agent), Parishin A exhibited a similar or even stronger inhibitory effect at higher concentrations (40 μM and 80 μM). The potency of Parishin A in preventing cell migration and invasion is thus comparable, if not superior, to the reference drug, highlighting its potential as a promising therapeutic candidate for OSCC treatment. These findings were supported by alterations in EMT markers, with E-cadherin levels rising and both N-cadherin and vimentin levels falling (Figure 4A,B). By inhibiting EMT, Parishin A effectively reduces the metastatic potential of OSCC cells, underscoring its role in preventing cancer spread, which is a major cause of cancer-related mortality. Given the critical role of EMT in metastasis, further experiments could focus on investigating the detailed molecular mechanisms through which Parishin A modulates EMT-related pathways, potentially leading to the development of new therapeutic strategies targeting cancer metastasis.

The PI3K/AKT/mTOR signaling pathway plays a vital role in regulating key cellular processes such as growth, proliferation, and survival, and is often dysregulated in various cancers, including OSCC [22]. This pathway is triggered when growth factors bind to receptor tyrosine kinases, leading to the phosphorylation and activation of PI3K [23]. Activated PI3K generates PIP3, which attracts AKT to the cell membrane, where it is then phosphorylated by PDK1 and mTORC2. Activated AKT subsequently phosphorylates various downstream targets, including mTOR, promoting cell growth, survival, and proliferation. Dysregulation of this pathway contributes to tumor progression and metastasis, making it a critical target for cancer therapy [24,25]. In our study, IHC and Western blot analyses revealed that Parishin A treatment decreases the phosphorylation levels of PI3K, AKT, and mTOR, whereas the total protein levels remain unchanged (Figure 5C,D). This indicates that Parishin A effectively inhibits the activation of the PI3K/AKT/mTOR pathway, thereby disrupting the signaling mechanisms essential for cancer cell maintenance. The activation level of the PI3K/AKT pathway is relatively low in normal cells, which contrasts sharply with its often-elevated activity in cancer cells [26]. In normal cells, the PI3K/AKT pathway primarily maintains basic cell survival and metabolic functions, while in cancer cells, it is hyperactivated to promote cell proliferation, inhibit apoptosis, and enhance metabolic activities [27]. This is also verified in our results (Figure 5C,D); this difference may explain the selective effects of Parishin A on normal versus cancer cells, further supporting its potential as an anti-cancer agent. The inhibition of this pathway by Parishin A is consistent with the observed reductions in cell viability, colony formation, migration, and invasion. By suppressing the PI3K/AKT/mTOR pathway, Parishin A reduces the signaling required for cancer cell proliferation and survival, leading to decreased tumor growth and potentially limiting metastasis. This suppression of the pathway likely also contributes to the inhibition of the EMT process, as AKT signaling is known to promote EMT through the regulation of various transcription factors and proteins involved in cell adhesion and motility [28]. The reliability of Parishin A as a candidate for additional experimental research is strengthened by its consistent inhibition of crucial cancer signaling pathways, indicating it is a promising molecule for further investigation in both preclinical and clinical contexts.

**Figure 6:**
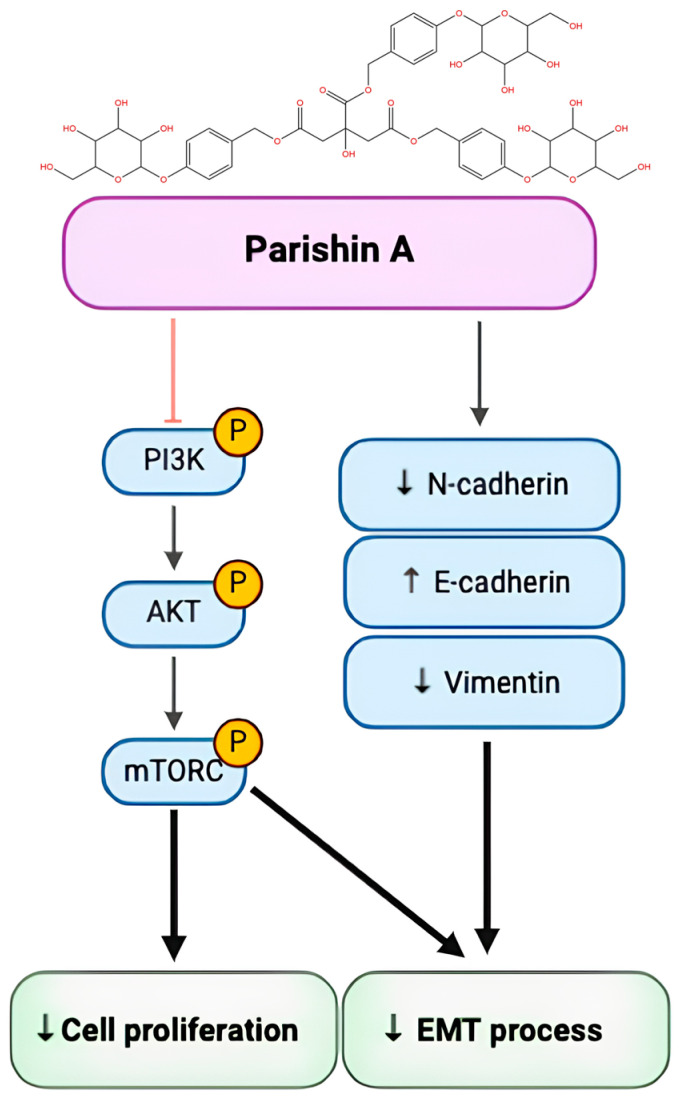
Parishin A suppresses OSCC cell growth and the EMT process by influencing the PI3K/AKT/mTOR signaling pathway.

In conclusion, Parishin A treatment increases the expression of E-cadherin while decreasing the expression of N-cadherin and vimentin, thereby inhibiting the EMT process. Concurrently, Parishin A suppresses the phosphorylation of PI3K, AKT, and mTOR, thereby decreasing cell proliferation. These findings illustrate the dual impact of Parishin A in inhibiting both cell proliferation and the EMT process (Figure 6). This provides a strong foundation for further research and development of Parishin A as a novel therapeutic agent for OSCC, with the potential to improve patient prognosis and reduce the burden of this aggressive cancer.

## 4. Materials and Methods

### 4.1. Ethical Statement

All protocols described herein were approved by the Institutional Review Board of Kyungpook National University (KNUDH-2022-07-02-00).

### 4.2. Reagents and Antibodies

Parishin A, with a purity greater than 98% (Harvey Biotech Co., Beijing, China), was dissolved in dimethyl sulfoxide (DMSO) at various working concentrations. Cisplatin (CDDP) was generously provided by Dr. Yong-Gun Kim from the Department of Periodontology, Kyungpook National University School of Dentistry, Daegu, Korea. The primary antibodies used in this study, obtained from Santa Cruz Biotechnology Inc. (Dallas, TX, USA), included E-cadherin (sc-21791), N-cadherin (sc-59987), vimentin (sc-6260), and β-actin (sc-47778). Additionally, antibodies for phosphorylated AKT (p-AKT; #9271), AKT (#9272), phosphorylated PI3K (p-PI3K; #17366), PI3K (#4249), phosphorylated mTOR (p-mTOR; #5536), and mTOR (#2983) were procured from Cell Signaling Technology (Danvers, USA).

### 4.3. Cell Culture

Using a previously described tissue explant technique [29], primary human gingival fibroblasts (HGnFs) were isolated from three healthy individuals under 35 years old with no systemic or periodontal conditions. These fibroblasts were derived from 1 mm tissue biopsies and cultured in Dulbecco’s Modified Eagle Medium (DMEM; Gibco™, Waltham, USA), supplemented with 10% fetal bovine serum (FBS; GenDEPOT, Katy, USA) and 1% penicillin/streptomycin (PS; Gibco™, Waltham, USA). The cells, which did not exceed seven passages, were deprived of nutrients by being cultured in medium with 0.3% FBS before experimental treatment. Human oral squamous cell carcinoma (OSCC) lines, YD-10B and Ca9-22, were provided by the Department of Oral Biology at Yonsei University, Seoul, Republic of Korea. These OSCC cell lines were maintained in DMEM with 10% FBS and 1% PS and incubated in a humidified chamber with 5% CO_2_ at 37 °C.

### 4.4. Cell Viability Assay

Cell viability was assessed using the Cell Counting Kit-8 (CCK-8) assay. YD-10B and Ca9-22 cells were seeded in 96-well plates at a density of 5 × 10^3^ cells per well and allowed to adhere overnight. The cells were then treated with PA at various concentrations (0, 10, 20, 40, 60, and 80 µM) for different time intervals (0, 24, 48, 72, and 96 h). After treatment, 10 µL of CCK-8 reagent was added to each well, and the plates were incubated for an additional 2 h at 37 °C. Absorbance was measured at 450 nm using a microplate reader.

### 4.5. Colony Formation Assay

The effect of PA on the colony-forming ability of OSCC cells was evaluated using agarose gel. YD-10B and Ca9-22 cells were plated in 6-well plates at a density of 500 cells per well and treated with various concentrations of PA (0, 10, 20, 40, 60, and 80 µM). A base layer of 0.6% agarose in DMEM with 10% FBS was first prepared. The cells were suspended in a top layer of 0.3% agarose in DMEM with 10% FBS and then overlaid onto the base layer. The cells were then incubated for 14 days to allow colony formation. Colonies were stained with 0.3% crystal violet and counted under a microscope.

### 4.6. Wound Healing Assay

The wound healing assay was used to evaluate the migratory ability of OSCC cells in response to PA treatment. YD-10B and Ca9-22 cells were seeded in 6-well plates at a density of 1 × 10^5^ cells per well and allowed to grow to near-confluence. A sterile 200 µL pipette tip was used to create a uniform scratch across the cell monolayer. The cells were then washed with phosphate-buffered saline (PBS) to remove detached cells and debris. Cells were treated with various concentrations of PA (0, 20, 40, and 80 µM) in serum-free DMEM. The initial wound width was recorded, and images of the wound area were captured at 0 and 12 h using an inverted microscope. The migratory distance of the cells was measured using the ImageJ software 1.54.

### 4.7. Cell Invasion Assay

Cell invasion was examined using a Matrigel invasion assay. Transwell inserts (8 µm pore size) were first coated with Matrigel (Corning Costar, Lowell, MA, USA). YD-10B and Ca9-22 cells were seeded in the upper chamber of the transwell inserts at a density of 1 × 10^5^ cells per well in serum-free DMEM. The lower chamber was filled with DMEM containing 10% FBS as a chemoattractant. After 48 h of incubation, non-invading cells were removed from the upper surface of the membrane, and the invading cells on the lower surface were fixed, stained with crystal violet, and counted under a microscope.

### 4.8. Western Blot Analysis

Cells were lysed in ethylenediaminetetraacetic acid (EDTA)-free RIPA II cell lysis buffer (1×) supplemented with Triton a protease inhibitor cocktail (100×). Protein concentrations were determined using the BCA protein assay kit (Thermo Fisher Scientific, Waltham, USA). Equal amounts of protein (30 µg) were separated by SDS-PAGE and transferred to PVDF membranes. Membranes were blocked with 5% non-fat milk in TBST and probed with primary antibodies. After incubation with HRP-conjugated secondary antibodies, signals were detected using an ECL detection system (Bio-Rad, Hercules, USA) and imaged with the ImageQuant LAS 500 System.

### 4.9. Tissue Array

The Fifth-Generation Tissue Array (T-BO-1-TARP) was acquired from the National Cancer Institute Array program. This array encompasses oral squamous cell carcinoma tissue samples along with marginal or paracancerous tissues. Specifically, it contains 50 cases of squamous cell carcinoma and 10 cases of adjacent normal or cancer-adjacent tissues, with a single core per case. Detailed parameters of the array are provided in Supplementary Table S1.

### 4.10. Immunohistochemistry Analysis

Immunohistochemistry (IHC) analyses were conducted using 4 μm thick paraffin-embedded tissue sections. The sections were blocked with 1% BSA and incubated overnight at 4 °C with the primary antibody p-PI3K (AF3242, Affinity Biosciences, Jiangsu, China). The tissue sections were then deparaffinized, rehydrated, and permeabilized with 0.5% Triton X-100 in PBS for 10 min. After three PBS washes, the sections were incubated with an appropriate secondary antibody. Protein targets were visualized using 3,3′-Diaminobenzidine (DAB) staining according to the manufacturer’s instructions. The sections were counterstained with hematoxylin for 2 min and imaged under a microscope, after which the results were analyzed and quantified using the Image-Pro Plus software (v.6.1) from Media Cybernetics.

### 4.11. Statistical Analysis

All experiments were carried out in triplicate, and the results are presented as the mean ± standard deviation (SD). Statistical analysis was conducted using SPSS software (version 23.0; IBM Inc., Chicago, IL, USA). Group differences were assessed using one-way analysis of variance (ANOVA), followed by Tukey’s post hoc test for multiple comparisons. A *p*-value of less than 0.05 was deemed statistically significant.

## Data Availability

Data are contained within the article and Supplementary Materials.

## Supplementary Materials

The following supporting information can be downloaded at: https://www.mdpi.com/article/10.3390/ph17101277/s1, Table S1. Clinicopathologic data of the 60 patients with OSCC.
