# Supplementary material for: Parishin A Inhibits Oral Squamous Cell Carcinoma via the AKT/mTOR Signaling Pathway
Source: Pharmaceuticals (Basel). 2024 Sep 26;17(10):1277. doi: 10.3390/ph17101277 (PMC11510427; doi:10.3390/ph17101277)
Supplement: Supplementary file 1 [file pharmaceuticals-17-01277-s001.zip › pharmaceuticals-3149899-supplementary.pdf]

Supplementary Table S1. Clinicopathologic data of the 60 patients with OSCC.

Description	Oral cavity cancer tissue array with normal oral cavity tissue, including pathology grade, TNM and clinical stage, 60 cases/60 cores, replacing OR601c
Total cases	60
Array panel	Oral cavity squamous cell carcinoma tissue microarray with normal oral cavity tissue, containing 50 cases squamous cell carcinoma and 10 cases adjacent normal or cancer adjacent tissue, single core per case

Detailed information of tissue array panel

No.	Age	Sex	Organ/Anatomic Site	Pathology diagnosis	Grade	TNM	Satge	Tissue ID.	Type
1	78	M	Tongue	Squamous cell carcinoma of tongue	1	T2N0M0	II	Doc022782	Malignant
2	40	M	Tongue	Squamous cell carcinoma of tongue	1	T2N0M0	II	Doc100435	Malignant
3	75	F	Tongue	Squamous cell carcinoma of tongue	1	T2N0M0	II	Doc100192	Malignant
4	35	F	Tongue	Squamous cell carcinoma of tongue	1	T2N0M0	II	Doc022134	Malignant
5	61	M	Tongue	Squamous cell carcinoma of tongue	1	T2N0M0	II	Doc090182	Malignant
6	41	F	Tongue	Squamous cell carcinoma of tongue	1	T2N0M0	II	Doc024253	Malignant
7	64	M	Tongue	Squamous cell carcinoma of right tongue	1	T2N2M0	IVA	Doc023998	Malignant
8	76	M	Tongue	Squamous cell carcinoma of tongue	1	T1N0M0	I	Doc031829	Malignant
9	50	F	Tongue	Squamous cell carcinoma of tongue	1	T2N0M0	II	Doc040310	Malignant
10	44	M	Tongue	Squamous cell carcinoma of tongue	1	T2N1M0	III	Doc090185	Malignant
11	53	F	Tongue	Squamous cell carcinoma of tongue	1	T1N0M0	I	Doc061514	Malignant
12	46	F	Tongue	Squamous cell carcinoma of tongue(skeletal muscle)	-	T2N0M0	II	Doc031255	Malignant
13	50	M	Tongue	Squamous cell carcinoma of root of tongue	1	T3N1M0	III	Doc050175	Malignant
14	36	F	Tongue	Squamous cell carcinoma of tongue	1	T1N0M0	I	Doc024234	Malignant
15	63	F	Tongue	Squamous cell carcinoma of tongue	1	T1N0M0	I	Doc031313	Malignant
16	46	M	Tongue	Squamous cell carcinoma of tongue	1	T2N0M0	II	Doc100401	Malignant
17	58	M	Tongue	Squamous cell carcinoma of tongue	1	T2N0M0	II	Doc110193	Malignant
18	64	M	Lip	Squamous cell carcinoma of lower lip	1	T1N0M0	I	Doc040556	Malignant
19	57	M	Lip	Squamous cell carcinoma of lower lip	1	T2N0M0	II	Doc110007	Malignant
20	61	M	Lip	Squamous cell carcinoma of lower lip	1	T1N0M0	I	Doc040354	Malignant
21	60	M	Gum	Squamous cell carcinoma of gum	1	T3N0M0	III	Doc100308	Malignant
22	60	M	Gum	Squamous cell carcinoma of gum	1	T1N0M0	I	Doc021673	Malignant
23	69	M	Gum	Squamous cell carcinoma of upper gum	1	T3N0M0	III	Doc080032	Malignant
24	53	M	Bucca cavioris	Squamous cell carcinoma of right bucca cavioris	1	T2N0M0	II	Doc023948	Malignant
25	55	M	Bucca cavioris	Squamous cell carcinoma of right bucca cavioris	1	T1N0M0	I	Doc023942	Malignant
26	58	M	Oral cavity	Squamous cell carcinoma of floor of mouth	1	T1N0M0	I	Doc024239	Malignant
27	63	M	Oral cavity	Squamous cell carcinoma	1	T1N0M0	I	Doc024257	Malignant
28	48	F	Tongue	Squamous cell carcinoma of tongue	1--2	T1N0M0	I	Doc031806	Malignant
29	80	M	Lip	Squamous cell carcinoma of lower lip	1--2	T1N0M0	I	Doc040320	Malignant
30	77	M	Oral cavity	Squamous cell carcinoma of floor of mouth	1--2	T2N0M0	II	Doc024238	Malignant
31	59	M	Tongue	Squamous cell carcinoma of tongue	2	T2N0M0	II	Doc090184	Malignant
32	77	F	Tongue	Squamous cell carcinoma of tongue	2	T1N0M0	I	Doc061028	Malignant
33	56	M	Tongue	Squamous cell carcinoma of root of tongue	2	T2N1M0	III	Doc080363	Malignant
34	60	M	Tongue	Squamous cell carcinoma of tongue	2	T2N1M0	III	Doc110126	Malignant
35	62	M	Tongue	Squamous cell carcinoma of tongue	2	T2N0M0	II	Doc110156	Malignant
36	67	F	Tongue	Squamous cell carcinoma of tongue	2	T2N0M0	II	Doc060099	Malignant
37	47	F	Tongue	Squamous cell carcinoma of tongue	2	T2N0M0	II	Doc030433	Malignant
38	37	M	Tongue	Squamous cell carcinoma of tongue	2	T2N1M0	III	Doc024045	Malignant
39	55	F	Tongue	Squamous cell carcinoma of tongue (sparse)	2	T2N0M0	II	Doc031727	Malignant
40	56	F	Bucca cavioris	Squamous cell carcinoma of left bucca cavioris	2	T2N0M0	II	Doc021328	Malignant
41	49	M	Bucca cavioris	Squamous cell carcinoma of right bucca cavioris	2	T1N0M0	I	Doc023950	Malignant
42	45	M	Bucca cavioris	Squamous cell carcinoma of right bucca cavioris	2	T2N0M0	II	Doc040434	Malignant
43	42	M	Bucca cavioris	Squamous cell carcinoma of bucca cavioris	2	T3N0M0	III	Doc110061	Malignant
44	44	M	Submaxilla	Squamous cell carcinoma of right submaxilla	2	T1N0M0	I	Doc110009	Malignant
45	40	F	Oral cavity	Squamous cell carcinoma of floor of mouth (sparse)	2	T2N0M0	II	Doc024246	Malignant
46	49	M	Bucca cavioris	Squamous cell carcinoma of left bucca cavioris	2	T1N0M0	I	Doc031444	Malignant
47	56	F	Oral cavity	Squamous cell carcinoma of floor of mouth	3	T2N0M0	II	Doc030020	Malignant
48	42	M	Bucca cavioris	Squamous cell carcinoma of bucca cavioris	3	T3N0M0	III	Doc110061	Malignant
49	87	F	Face	Squamous cell carcinoma of left face	3	T2N0M0	II	Doc100187	Malignant
50	50	M	Gum	Squamous cell carcinoma of gum	3	T2N0M0	II	Doc060153	Malignant
51	76	M	Oral cavity	Adjacent normal tongue tissue	-	-	-	Doc031829	NAT
52	30	M	Oral cavity	Adjacent normal tongue tissue	-	-	-	Doc040634	NAT
53	50	M	Oral cavity	Adjacent normal tongue tissue	-	-	-	Doc062627	NAT
54	2. Mon	M	Oral cavity	Adjacent normal tongue tissue	-	-	-	Doc031954	NAT
55	49	M	Oral cavity	Adjacent normal tongue tissue	-	-	-	Doc030573	NAT
56	42	M	Oral cavity	Adjacent normal tongue tissue	-	-	-	Doc130443	NAT
57	50	F	Oral cavity	Adjacent normal tongue tissue	-	-	-	Doc120119	NAT
58	83	F	Oral cavity	Cancer adjecent maxilla tissue	-	-	-	Doc070413	AT
59	56	M	Oral cavity	Cancer adjecent maxilla tissue	-	-	-	Doc070392	AT
60	62	M	Oral cavity	Cancer adjecent maxilla tissue	-	-	-	Doc040194	AT
